# Body mass index and association with use of and distance from places for physical activity and active leisure among schoolchildren in Brazil. Cross-sectional study

**DOI:** 10.1590/1516-3180.2017.0347020118

**Published:** 2018-06-18

**Authors:** Camila Elizandra Rossi, Elizabeth Nappi Correa, Janaina das Neves, Cristine Garcia Gabriel, Jucemar Benedet, Cassiano Ricardo Rech, Francisco de Assis Guedes de Vasconcelos

**Affiliations:** I MSc. Doctoral Student, Universidade Federal de Santa Catarina (UFSC), Florianópolis (SC), Brazil.; II MD, PhD. Researcher, Department of Nutrition, Universidade Federal de Santa Catarina (UFSC), Florianópolis (SC), Brazil.; III MD, PhD. Researcher, Postgraduate Program on Nutrition, Department of Nutrition, Universidade Federal de Santa Catarina (UFSC), Florianópolis (SC), Brazil.; IV MD, PhD. Researcher, Postgraduate Program on Nutrition, Department of Nutrition, Universidade Federal de Santa Catarina (UFSC), Florianópolis (SC), Brazil.; V MD, PhD. Researcher, Postgraduate Program on Physical Education, Department of Sports, Universidade Federal de Santa Catarina (UFSC), Florianopolis (SC), Brazil.; VI MD, PhD. Researcher, Postgraduate Program on Physical Education, Department of Sports, Universidade Federal de Santa Catarina (UFSC), Florianópolis (SC), Brazil.; VII MD, PhD. Researcher, Postgraduate Program on Nutrition, Universidade Federal de Santa Catarina (UFSC), Florianópolis (SC), Brazil.

**Keywords:** Environment and public health, Socioeconomic factors, Overweight, Youth

## Abstract

**BACKGROUND:**

We evaluated associations between use of public places for physical activity and active leisure (PAAL) and their distances from subjects’ homes and indicators of overweight and obesity, among schoolchildren from different socioeconomic levels, in the city of Florianópolis, Brazil.

**DESIGN AND SETTING:**

Cross-sectional study conducted on a sample of 2,152 schoolchildren aged 7 to 14 years, enrolled at 30 public and private schools.

**METHODS:**

The exposure variables were the use of public places for PAAL in the neighborhood and their distance from schoolchildren’s homes. The outcomes were body mass index (BMI) and waist circumference (WC). Univariate and multivariate linear regression analyses were conducted according to income strata.

**RESULTS:**

Among the schoolchildren from low-income families, living closer to parks/playgrounds was associated with lower BMI (β = -2.15; 95% confidence interval, CI = -2.53; -1.77) and lower WC (β = -0.11 95% CI = -0.17; -0.05), while living at these distances from football pitches was associated with higher BMI (β = 1.73; 95% CI = 0.31; 3.15) and larger WC measurements (β = 0.03; 95% CI = 0.005; 0.14). Among the schoolchildren in low-income groups, living at an intermediate distance from beaches was associated with lower BMI (β = -1.10; 95% CI = -1.61; -0.59).

**CONCLUSION:**

Living closer to parks/playgrounds was associated with lower BMI and WC among schoolchildren from low-income families. Living closer to football pitches was associated with higher BMI and WC among these schoolchildren. Living at intermediate distances from beaches was associated with lower BMI among these schoolchildren.

## INTRODUCTION

There is evidence showing increasing prevalence of overweight and obesity among children and adolescents in high, medium and low-income countries.^1^ Similar trends were observed in Florianópolis, capital of the Brazilian state of Santa Catarina, during the period from 2002 to 2007, among 7 to 10-year-old children,^2^ and from 2007 to 2012, among 7 to 14-year-old children and adolescents.^3^ In addition, in 2007 and 2008, the prevalence of abdominal obesity among 6 to 10-year-old schoolchildren in the state of Santa Catarina was observed to be 4.9%.^4^

Studies have shown that the availability of places for physical activity and active leisure (PAAL) close to home makes it more likely that these facilities will be used more often, because of ease of access.^5^ Children and adolescents who report living close to such places tend to have lower body mass index (BMI) and lower values for other measurements of obesity.^6^^,^^7^ It has also been indicated in the literature that there are differences in the patterns of usage of neighborhood facilities when different socioeconomic strata are investigated, whether assessed at the family^8^ or area level (the latter based on area of residence).^9^

However, the majority of published studies evaluating individuals of school age have been conducted in high-income countries, located in the northern hemisphere. Consequently, there is a lack of clear evidence regarding associations between measurements of overweight and obesity among children and adolescents living in middle-income and medium-to-high income countries and access to facilities for PAAL in the environs of their homes.^5^^,^^10^^,^^11^ Another gap in the literature is that the studies have assessed the environmental availability of public spaces in general, without analyzing different types of facilities separately or their relationships with adiposity-related outcomes.^6^^,^^12^^,^^13^^,^^14^

Florianópolis is the capital of the Brazilian state of Santa Catarina, which is located in the country’s southern administrative region. In 2016, the municipal district had a population density of 707.4 inhabitants/km^2^.^15^ Although the city of Florianópolis has a very high human development index (HDI; 0.847),^16^ it also has an elevated Gini index of 0.5474 (the closer this index is to 1, the greater the social inequalities between residents are),^17^ which might reflect differences in access to places for PAAL between wealthy and underprivileged areas.

The objective of this study was therefore to investigate associations between use of public places for PAAL, and their distance from subjects’ homes, and indicators of overweight and obesity among 7 to 14-year-old schoolchildren from different socioeconomic levels, in the city of Florianópolis, Brazil.

## METHODS

### Ethics

This study was approved by the Human Research Ethics Committee at the Universidade Federal de Santa Catarina, under review process no. 120,341/2012. The guardians of all schoolchildren selected for the study were sent free and informed consent forms that they needed to sign before the children could be included in the study.

### Study design and participants

This was a cross-sectional study based on a probabilistic sample of 2,506 schoolchildren aged 7-14 years who were enrolled at public or private schools in Florianópolis. The sample was selected by means of clusters, according to: the municipal district’s administrative regions; the type of school; the age group; and the number of students enrolled in each school. This procedure aimed to ensure that the sample was representative both of the regions in which the population lives and of the variability of income in the population. The sampling methods have been described in greater detail elsewhere.^18^^,^^19^

Based on the prevalence rates of the exposure variables and of each outcome, and considering a study power of 80%, a 95% confidence level, a 10% sample size margin to allow for confounding factors and a design effect of 1.8, this study had sufficient power to: a) detect that prevalence ratios of 0.82 to 0.85 would be protective factors and 1.18 to 1.23 would be risk factors for overweight/obesity; and b) detect that prevalence ratios of 0.50 to 0.60 would be protective factors and 1.68 to 2.01 would be risk factors for abdominal obesity.

### Data collection

#### Study exposure variables

The schoolchildren were given self-report questionnaires, created for this study, which they and/or their parents/guardians answered. The questions included items asking about the frequency of use of, and perceived distance from home to places for physical activity and active leisure. These were chosen based on findings reported in the scientific literature that discusses these different types of places.^20^^,^^21^^,^^22^^,^^23^^,^^24^^,^^25^ Four types of places for PAAL were investigated regarding their frequency of use and perceived distance from home, as follows: beaches, parks/playgrounds, sports courts and football (soccer) pitches. These data were coded as categorical polytomous variables (used weekly, used fortnightly, used monthly, used rarely and never used).

In a nationwide study conducted in Brazil, on 74,589 adolescents aged 12 to 17 years, the subjects who were considered physically active were those for whom the duration of leisure-time physical activity was ≥ 300 minutes per week.^26^ In this light, and taking into account the possibility that each student might use several places in the vicinity of their homes, the variables of the present study regarding use of places were then re-categorized into two groups: did use them (covering weekly and fortnightly) and did not use them (combining used rarely, used monthly and never used).

The perceived distance from the family home to each type of place was surveyed in terms of the time taken to walk the distance in minutes. The responses were categorized as up to 10 minutes, from 11 to 19 minutes and 20 minutes or more, on the assumption that places that took up to 10 minutes to reach on foot were close to the home (approximately 800 meters) and could therefore be accessed actively, without the need for motorized transportation.^27^

The questionnaire answered by the children and their parents also contained questions on monthly family income and mothers’ and fathers’ educational levels. The parents’ educational levels were classified into three categories (incomplete high school, complete high school, complete higher education). The schoolchildren’s ages were analyzed both as a continuous variable and dichotomously in two categories according to the sampling frame applied (7 to 10 or 11 to 14 years of age), and the variable of type of school was divided into two categories (public or private). Monthly family incomes reported in Brazilian reais (R$) were collected as a continuous variable and were then used to stratify the sample in terciles (high, medium or low-income families), in order to observe whether physical activity environments were associated with the outcome measurements differently for distinct socioeconomic strata. The terciles of monthly income that were used to separate the sample into low, medium and high-income strata were R$ < 1,577 (first tercile), R$ 1,577 to 3,001 (second tercile) and R$ > 3,001 (third tercile).

#### Study outcome variables

Weight and height data were collected objectively by researchers who had been duly trained in accordance with the technical standards recommended by the World Health Organization (WHO).^28^ The absolute intra-examiner technical error of measurement (TEM) that was considered acceptable was twice that of the gold-standard anthropometrist, while the absolute inter-examiner TEM that was considered acceptable was three times the experienced anthropometrist’s TEM.^29^

BMI, as evaluated according to the WHO criteria,^30^ has shown high sensitivity (92.5%) for detecting excess body fatness in schoolchildren aged 7-10 years living in Florianópolis.^31^ Hence, we defined overweight as BMI for age and sex ≥ +1 and < +2 z-scores and obesity as BMI for age and sex ≥ + 2 z-scores.^30^ In addition, we evaluated waist circumference (WC) in our sample. This measurement was taken at least twice for each schoolchild. These data were used as continuous variables, in cm, in the analyses on associations. WC values were categorized using the criterion for abdominal obesity proposed by Fernandez et al.^32^ (percentile ≥ 90 for age and sex as the cutoff point) to observe its prevalence in the sample.

### Statistical analyses

Data on the variables of use of places for PAAL and perceived distance from these places were taken to be the primary exposures. Their associations with the two continuous outcome variables BMI (in kilograms divided by meters squared) and WC (in centimeters) were tested using univariate and multivariate linear regression, with estimation of β coefficients and 95% confidence intervals (95% CI).

Exposure variables with P-values ≤ 0.20 for univariate associations with outcomes were entered into a multivariate model with forward selection in the order of their strength of association (the higher the P-value was, the earlier the variable was included in the multivariate model). Interactions between the environmental factors and the outcome were tested for sex and age strata first, prior to income stratification, and no differences relating to sex or age strata were observed in these correlations (data not shown).

A 5% significance level was used for hypothesis testing, considering type I error, and null hypotheses were rejected when the p-value was less than the type I error value. The *svy* command available in the Stata 13.0 software was used to account for the sample weights of each individual. When multivariate models had been constructed, their goodness of fit was analyzed using the Bartlett test (homogeneity of variance) for qualitative variables. Models were defined as presenting a good fit when they had P-values > 0.05.

## RESULTS

The study investigated 2,506 schoolchildren. Valid BMI data were obtained from 2,484, and there was at least one valid WC measurement for 2,480. Valid family income data were obtained in relation to 2,152 (85.9% of the sample).

Table 1 lists the characteristics of the whole sample and those of the sample broken down according to family income strata. Overall, the prevalence of overweight was 21.5% and the prevalence of obesity was 12.7%, thus showing that more than one third of the schoolchildren had excess body weight. Abdominal obesity was detected in 5.0% of those assessed. There were no significant differences in the mean body mass index or mean waist circumference between the income strata. In relation to the parents’ educational level, it was observed that among the schoolchildren from the high-income stratum, more of both the mothers and the fathers had completed undergraduate university education (Table 1).

**Table 1:** Descriptive characteristics of the sample of 7 to 14-year-old schoolchildren, stratified according to monthly family income, Florianópolis, Santa Catarina, Brazil, 2012-2013

Variables	Categories	Total (n = 2,506)		Low income (n = 718)		Medium income (n = 736)		High income (n = 698)		P-value^b^
		n	%	n	%^a^	n	%^a^	n	%^a^	
Sex	Female	1,334	56.5	402	58.0	370	54.0	362	56.7	0.640
	Male	1,172	43.5	316	42.0	366	46.0	336	43.3	
Age (years)	7 to 10	1,530	61.1	436	61.2	473	63.9	415	63.0	0.589
	11 to 14	976	38.9	282	38.8	263	36.1	283	37.0	
Type of school	Public	1,637	65.3	672	93.9	585	80.7	201	26.2	< 0.001
	Private	869	34.7	46	6.1	151	19.3	497	73.8	
BMI (n = 2,484)	(Mean; SD)	18.60	3.55	18.72	3.80	18.74	3.71	18.44	3.24	0.616
	Overweight^*^	511	21.5	469	65.6	473	65.6	467	65.7	0.995
	Obese^†^	315	12.7	241	34.4	256	34.4	226	34.3	
Waist circumference (n = 2,480)	(Mean; SD)	61.92	8.53	61.95	3.89	61.76	3.99	62.10	3.99	0.718
Abdominal obesity (n = 2,480)	Yes^‡^	134	5.0	51	6.6	48	5.5	25	3.7	0.198
Mother’s educational level (n = 2,389)	Incomplete high school	851	33.3	443	63.5	272	35.3	65	7.6	< 0.001
	Complete high school	857	37.5	210	32.5	336	49.1	210	32.7	
	Complete higher education	681	29.2	32	4.0	115	15.6	416	59.7	
Father’s educational level (n = 2,086)	Incomplete high school	806	35.6	364	62.6	295	44.1	83	11.4	0.002
	Complete high school	710	35.3	144	31.0	268	44.7	215	30.0	
	Complete higher education	570	29.1	39	6.4	80	11.2	343	58.6	
Uses beaches (n = 2,382)	Yes	745	32.9	199	30.2	200	26.5	223	35.7	0.456
	No	1,637	67.1	489	69.8	522	73.5	462	64.3	
Uses parks/ playgrounds (n = 2,342)	Yes	642	27.5	169	23.2	183	24.1	205	32.6	0.045
	No	1,700	72.5	501	76.8	523	75.9	476	67.4	
Uses sports courts (n = 2,336)	Yes	1,100	45.3	325	47.4	322	41.3	309	45.0	0.288
	No	1,236	54.7	345	52.6	388	58.7	366	55.0	
Uses football pitches (n = 2,341)	Yes	661	25.4	209	26.1	214	25.2	159	23.5	0.836
	No	1,680	74.6	461	73.9	501	74.8	517	76.5	
Distance to parks/ playgrounds (minutes) (n = 1,830)	1-10	776	42.4	173	31.0	221	43.7	268	56.0	0.101
	11-19	419	22.9	150	30.3	126	19.5	103	14.3	
	≥ 20	635	34.7	178	38.7	202	36.8	179	29.6	
Distance to sports courts (minutes) (n = 1,508)	1-10	698	46.3	173	39.7	207	44.4	224	53.3	0.132
	11-19	372	24.6	127	30.0	114	20.7	92	22.2	
	≥ 20	438	29.1	117	30.3	157	34.9	111	24.5	
Distance to football pitches (minutes) (n = 1,244)	1-10	538	43.3	141	35.6	172	38.3	161	49.5	0.002
	11-19	306	24.6	101	29.7	106	31.3	71	21.1	
	≥ 20	400	32.1	123	34.7	128	30.4	97	29.4	
Distance to beaches (minutes) (n = 2,072)	1-10	476	23.0	122	20.4	138	21.7	144	20.9	0.829
	11-19	360	17.3	109	21.3	111	20.0	108	17.8	
	≥ 20	1,236	59.7	359	58.3	368	58.3	368	61.3	

BMI = body mass index; *overweight 95% CI = 16.7-27.3%; ^†^obese 95% CI = 11.0-14.5%; ^‡^abdominal obesity 95% CI = 3.4-7.3%; ^a^percentage values take into account the design effect (svy command); ^b^P-value significant at 5% for Pearson’s chi-square test.

The data on the use of each of the different types of public places for PAAL showed that sports courts were the most popular, followed by beaches, parks/playgrounds and, finally, football pitches. Differences in the use of these places according to income strata were observed only in relation to parks/playgrounds, which were used more frequently by schoolchildren in the high-income group. Schoolchildren from the highest income stratum were the ones who most frequently lived closer to football pitches (Table 1).

The frequencies of use of all places for PAAL were significantly and progressively higher when places were near home (Table 2). Multivariate analyses showed that schoolchildren in the low-income group who live at intermediate and closer distances from parks/playgrounds had lower BMI values (β = -1.96; 95% CI = -3.45; -0.47; and β = -2.15; 95% CI = -2.53; -1.77, respectively), compared with schoolchildren living far from these facilities. Schoolchildren from the low-income stratum who lived at an intermediate distance from beaches also presented lower values of BMI (β = -1.10; 95% CI = -1.61; -0.59). These associations were also observed between higher values of BMI and intermediate and closer distances from football pitches (β = 1.67; 95% CI = 0.72; 2.62; and β = 1.73; 95% CI = 0.31; 3.15, respectively) (Table 3).

**Table 2:** Frequency of schoolchildren using places for PAAL according to perceived distances from the places to home. Florianópolis, Santa Catarina, Brazil, 2012-2013

Frequency of use of places for PAAL	Perceived distance from home in time walking (minutes)						P-value*
	1 - 10		11 - 20		> 21		
Uses beaches (n = 2,069)	n	%	n	%	n	%	
Yes	300	67.2	150	42.6	277	23.2	< 0.000
No	174	32.8	210	57.4	958	76.8	
Uses parks/ playgrounds (n = 1,826)							
Yes	378	48.1	139	32.6	114	16.2	< 0.000
No	395	51.9	279	67.4	521	83.8	
Uses sports courts (n = 1,505)							
Yes	538	75.4	257	68.2	232	50.9	< 0.000
No	159	24.6	113	31.8	206	49.1	
Uses football pitches (n = 1,237)							
Yes	326	59.4	160	49.1	147	36.0	< 0.000
No	208	40.6	145	50.9	251	64.0	

*Tendency from chi-square test.

**Table 3:** Crude and adjusted analyses on association between use of public places for physical activity and their distances from homes and body mass index, according to family income strata, among 7 to 14-year-old schoolchildren living in Florianópolis, Santa Catarina, Brazil, 2012-2013

Environmental variables	Low income				Medium income				High income			
	Crude analyses^a^		Adjusted analyses*^,a,b^		Crude analyses^a^		Adjusted analyses*^,a,b^		Crude analyses^a^		Adjusted analyses*^,a,b^	
	β	95% CI	β	95% CI	β	95% CI	β	95% CI	β	95% CI	β	95% CI
Uses beaches												
No	0.00		-	-	0.00		0.00		0.00		0.00	
Yes	-0.01	-0.71; 0.69	-	-	0.25	-0.44; 0.95	0.21	-1.11; 1.53	-0.33	-0.55; -0.09	-0.10	-1.69; 1.49
Uses parks/ playgrounds												
No	0.00		0.00		0.00		0.00		0.00		-	-
Yes	-0.39	-1.12; 0.32	0.61	-0.79; 2.00	-0.45	-1.67; 0.77	-0.45	-2.01; 1.10	-0.56	-2.52; 1.40	-	-
Uses sports courts												
No	0.00		0.00		0.00		0.00		0.00		0.00	
Yes	0.58	-0.23; 1.40	0.79	-1.41; 2.99	0.008	-0.94; 0.96	-0.44	-2.32; 1.43	0.88	-0.58; 2.34	0.08	-3.23; 3.39
Uses football pitches												
No	0.00		-	-	0.00		0.00		0.00		-	
Yes	0.59	-0.48; 1.67	-	-	-0.08	-1.39; 1.22	-0.15	-1.95; 1.65	1.12	-0.34; 2.57	-	-
Distance to beaches (minutes)												
1-10	1.08	-0.49; 2.67	0.94	-1.92; 3.80	-0.25	-0.59; 0.08	-0.65	-1.60; 0.31	0.13	-0.11; 0.38	-	-
11-19	-0.02	-1.31; 1.26	-1.10	-1.61; -0.59	-0.58	-2.05; 0.89	-0.81	-2.36; 0.75	-0.14	-1.50; 1.22	-	-
≥ 20	0.00		0.00		0.00		0.00		0.00		-	-
Distance to parks/playgrounds (minutes)												
1-10	-0.77	-1.92; 0.38	-2.15	-2.53; -1.77	-0.46	-1.65; 0.73	-	-	0.78	0.44; 1.12	1.11	-0.12; 2.34
11-19	-1.52	-3.29; 0.24	-1.96	-3.45; -0.47	0.10	-1.36; 1.56	-	-	0.86	-1.00; 2.72	0.07	-1.20; 1.35
≥ 20	0.00		0.00	-	0.00		-	-	0.00		0.00	
Distance to sports courts (minutes)												
1-10	0.93	-0.85; 2.71	-	-	0.06	-1.11; 1.23	0.41	-0.40; 1.23	0.10	-2.05; 2.27	-	-
11-19	0.34	-2.00; 2.69	-	-	0.02	-1.42; 1.46	0.13	-1.31; 1.55	-0.11	-3.43; 3.21	-	-
≥ 20	0.00		-	-	0.00		0.00		0.00		-	-
Distance to football pitches (minutes)												
1-10	1.30	0.01; 2.59	1.73	0.31; 3.15	-0.09	-1.53; 1.33	-	-	0.81	0.38; 1.23	-0.15	-0.68; 0.37
11-19	0.53	0.18; 0.88	1.67	0.72; 2.62	0.19	-0.98; 1.35	-	-	0.67	-1.02; 2.37	-0.01	-1.35; 1.32
≥ 20	0.00		0.00		0.00		-	-	0.00		0.00	

*Multivariate models were controlled for the continuous variable of schoolchildren’s age; ^a^P-value significant at 5%; ^b^Variables of use of and distance from football pitches presented collinearity.

Table 4 shows that the same association that was observed between distance from home to parks/playgrounds and BMI was once again present in relation to the WC of schoolchildren in the low-income group (β = -0.11; 95% CI = -0.17; -0.05). For schoolchildren living 11-19 minutes away from football pitches, an association with WC was also observed (β = 0.03; 95% CI = 0.005; 0.14).

**Table 4:** Crude and adjusted analysis on association between use of public places for physical activity and their distances from homes and waist circumference, according to family income strata, among 7 to 14-year-old schoolchildren living in Florianópolis, Santa Catarina, Brazil, 2012-2013

Environmental variables	Low income				Medium income				High income			
	Crude analyses^a^		Adjusted analyses*^,a,b^		Crude analyses^a^		Adjusted analyses*^,a,b^		Crude analyses^a^		Adjusted analyses*^,a,b^	
	β	95% CI	β	95% CI	β	95% CI	β	95% CI	β	95% CI	β	95% CI
Uses beaches												
No	0.00		0.00	-	0.00		0.00		0.00		-	-
Yes	-0.44	-1.39; 0.51	-	-	0.62	-0.56; 1.80	-0.02	-0.26; 0.22	-0.86	-2.57; 0.83	-	-
Uses parks/ playgrounds												
No	0.00		-	-	0.00		0.00		0.00		0.00	
Yes	-1.05	-2.07; -0.03	0.008	-0.11; 0.13	-0.81	-1.80; 0.16	0.02	-0.10; 0.15	-1.49	-2.18; -0.80	-0.23	-0.48; 0.03
Uses sports courts												
No	0.00		0.00		0.00		-	-	0.00		0.00	
Yes	0.47	-0.14; 1.08	0.08	-0.12; 0.29	0.68	-2.11; 3.48	-	-	0.91	0.38; 1.44	0.06	-0.16; 0.29
Uses football pitches												
No	0.00		-	-	0.00		0.00		0.00		0.00	
Yes	0.63	-0.92; 2.17	-	-	1.19	0.76; 1.62	0.05	-0.07; 0.18	1.17	-0.61; 2.95	0.04	-0.05; 0.15
Distance to beaches (minutes)												
1-10	0.16	-0.44; 0.78	-	-	-0.08	-1.86; 1.69	-	-	-0.61	-2.42; 1.19	-	-
11-19	0.73	-2.02; 3.49	-	-	-0.61	-1.99; 0.77	-	-	-0.38	-2.38; 1.60	-	-
≥ 20	0.00		-	-	0.00		0.00		0.00		-	-
Distance to parks/playgrounds (minutes)												
1-10	-0.15	-1.24; 0.93	-0.07	-0.27; 0.12	-0.03	-2.00; 1.93	-	-	0.21	-1.30; 1.74	-	-
11-19	-1.23	-2.19; -0.26	-0.11	-0.17; -0.05	0.73	-2.26; 3.73	-	-	0.47	-1.78; 2.73	-	-
≥ 20	0.00		0.00		0.00		0.00		0.00		-	-
Distance to sports courts (minutes)												
1-10	0.76	-1.44; 2.97	-	-	0.06	-1.62; 1.74	-	-	1.34	0.46; 2.22	-0.18	-0.44; 0.07
11-19	-0.24	-1.03; 0.54	-	-	0.12	-2.34; 2.58	-	-	2.54	0.61; 4.50	-0.20	-0.40; 0.00
≥ 20	0.00		-	-	0.00		0.00		0.00		0.00	
Distance to football pitches (minutes)												
1-10	0.48	-1.36; 2.33	0.07	-0.17; 0.25	0.26	-0.25; 0.78	-	-	1.10	-0.03; 2.24	-	-
11-19	0.36	-2.21; 2.93	0.03	0.005; 0.14	-0.16	-1.44; 1.11	-	-	2.27	1.17; 3.37	-	-
≥ 20	0.00		0.00		0.00		-	-	0.00		-	-

*Multivariate models were controlled for the continuous variable of schoolchildren’s age; ^a^P-value significant at 5%; ^b^Variables of use of and distance from football pitches presented collinearity.

## DISCUSSION

This study analyzed the use of public places for PAAL and their distances from homes, and their associations with indicators of overweight/obesity among 7 to 14-year-old schoolchildren living in Florianópolis. The main findings were that associations existed in the stratum of schoolchildren in the low-income group between lower distances from parks/playgrounds and lower BMI and WC values; between lower distances from football pitches and higher BMI and WC values; and between living at an intermediate distance from beaches and lower values of BMI.

With regard to associations between indicators of overweight/obesity and socioeconomic characteristics, Boing and Subramanian^33^ assessed a population of a different age in Florianópolis. Their study enrolled 1,720 adults in 2009 and 2010, and it was observed that the BMI of women living in environments where educational levels were lower was 1.12 kg/m^2^ higher than the same index among female residents of areas with high educational levels (P < 0.05). Since the schoolchildren of our sample who were from low-income families had less-educated parents (Table 1), it is reasonable to assume that these schoolchildren from low-income families also lived in areas where incomes and educational levels were lower, thus raising the hypothesis that other variables relating to inequalities in the economic environment (such as availability of safe places for physical activity and existence of pedestrian-friendly environments in residential areas^9^^,^^34^) may be mediating overweight/obesity. Similar results have also been observed in other countries, albeit high-income countries. Lakes and Burkart^35^ assessed 28,159 children aged 5 to 6 years who were living in Berlin and observed that an increase of one point (on a scale from 4 to 8) in a family’s social index (relating to socioeconomic level) resulted in a 68.5% reduction in overweight.

Concerning the proximity of homes to parks/playgrounds and the association of this factor with both outcomes in the low-income family stratum, Hsieh et al.^36^ assessed Hispanic girls in Los Angeles, United States, who would be expected to have lower incomes than non-Hispanic residents. They found that the level of body fat was 1.4% lower among those who lived in neighborhoods with more than three acres of space reserved for parks. The higher density of parks around adolescents’ schools in Taiwan was also associated with lower values for waist circumference among boys.^37^

In relation to the association between intermediate distance from beaches to homes and lower BMI, Abbot et al.^38^ found similar results among 1,819 women (aged 18-66) living in Melbourne, Australia. The presence of a coastline within 2 km of less educated women’s homes explained 10.1% of the education-BMI relationship. These results suggest that beaches are also a good option for exercise, even among children and adults from low-income families.

In contrast with what was observed for parks/playgrounds, use of football pitches had a positive relationship with BMI and WC among low-income schoolchildren. These findings suggest reverse causality, thus indicating that low-income schoolchildren who have abdominal obesity use these places to exercise more. In fact, in Córdoba (Argentina) Lavin-Fueyo and Berra^39^ observed that the places for physical activity that schoolchildren from peripheral underprivileged neighborhoods used most were parks/public squares, in the first place, followed by football pitches. Another reason why we found a positive relationship between these variables could be that children and adolescent habitually go to football pitches to watch games, and this does not contribute towards improving their energy expenditure.

In our study, in the medium and higher-income strata, none of the environmental variables were associated with the outcomes in the adjusted analyses. One possible explanation for this is that in this socioeconomic stratum, other physical activity options, especially those that are supervised and consequently are not free of charge, may be used instead of public options. Bürgi et al.^9^ in Zurich, Switzerland, observed that children living in neighborhoods with higher socioeconomic status did the majority of their moderate to intense physical activities in schools other than their own, possibly through taking part in paid-for exercise options.

It is also interesting to note that even though the variable of the distance from places for physical activity was associated with both outcomes, the variable of “use” of the same type of places was not statistically significant in any of the income strata in our study. In our sample, we found a significant positive relationship between frequency of use of places and their proximity to schoolchildren’s homes (Table 2). Lavin-Fueyo et al.^40^ used the same approach as in our study, to investigate the use of these places and their distances from homes. They investigated 1,777 children aged 9 to 11 years in the city of Córdoba, Argentina, in 2011, and also found that use of parks was associated with their proximity to schoolchildren’s homes but was not associated with increases in the amount of physical activity. Among 22,889 adults evaluated in neighborhoods of Yorkshire, England, greater availability of parks within a distance of 2 km from home were associated both with lower BMI values and with lower prevalence of obesity.^41^ In Louisiana, United States, the body profile of 909 women and their children was evaluated and it was found that living in a neighborhood with less provision of parks, playgrounds and other recreational places was significantly associated both with higher BMI and with larger WC, after adjusting for covariates.^42^ These findings might indicate that the use of public spaces and open places for physical activities would be more frequent if these places are closer to home, and that maybe it is necessary to use them aiming to practice non-sedentary activities more than once a week, for there to be any positive effects regarding the intensity of physical activity and consequently regarding body profile. Such results are shown more frequently in adulthood.

The present study indicates that future analyses on the influence of the environment on physical activity and active leisure among children and adolescents living in medium-to-high income countries such as Brazil should take into account the economic inequalities affecting these populations, both at the family and at the environmental level. One strong point of the present study is the fact that the sample was probabilistic and randomized, with a sample that was representative of schools in all five geographical regions in the municipal district studied. The weighting effect of each person in the sample was also taken into account (svy command), which minimized bias in the analysis on variables for which there were fewer responses. Interactions between the environmental variables were also analyzed (chi-square analyses), which reduced bias due to collinearity in multivariate models (we found collinearity between the variables of use of football pitches and distance from home to these places).

The primary limitation of this study was its cross-sectional design, which means that additional evidence is needed to support the findings. In addition, our study may have been affected by a cause-effect relationship among the variables, such that some schoolchildren who were using places for physical activity may have been doing so as part of a treatment for obesity. A situation of this nature would possibly hide a previously existing association with high body mass index and high measurements of waist circumference. Furthermore, we did not assess data on physical activity levels and food intake, because several variables could not be properly fitted into the multivariate models. Moreover, the correlation between the self-reported measurements of distances from schoolchildren’s homes to the places with exercise facilities and objective measurements may not have been good. In such a situation, further studies regarding the feasibility of self-reported measurements for this issue would be required.

Our data indicate that there is a need to evaluate the relationship between the proximity of homes to places for PAAL and measurements of adiposity among the schoolchildren of Florianópolis in longitudinal studies, in order to confirm whether there is any direct relationship between the variables. If the findings from the present study are confirmed, it can be recommended that the public authorities responsible for urban planning of municipal districts should consider the need for creation of free public places for PAAL, especially in economically underprivileged areas, in order to encourage active behavior among their residents and prevent the emergence of overweight and obesity among children and adolescents in low-income groups.

## CONCLUSION

This study identified a significant association between proximity of parks/playgrounds to homes and lower BMI and WC values, and an association between short distances from homes to football pitches and higher BMI and WC among schoolchildren in low-income groups who were living in Florianópolis. An intermediate distance from homes to beaches was also associated with lower BMI values among schoolchildren from low-income families.
